# *Rauwolfia vomitoria* extract suppresses benign prostatic hyperplasia by inducing autophagic apoptosis through endoplasmic reticulum stress

**DOI:** 10.1186/s12906-022-03610-4

**Published:** 2022-05-05

**Authors:** Guifang Huang, Xiao He, Zesheng Xue, Yiming Long, Jiakuan Liu, Jinming Cai, Pengfei Tang, Bangmin Han, Bing Shen, Ruimin Huang, Jun Yan

**Affiliations:** 1 School of Chinese Materia Medica, Nanjing University of Chinese Medicine, 138 Xianlin Avenue, Nanjing, 210023 Jiangsu China; 2 Shanghai Institute of Materia Medica, Chinese Academy of Sciences, 555 Zuchongzhi Road, Shanghai, 201203 China; 3 Model Animal Research Center of Nanjing University, 12 Xuefu Road, Nanjing, 210061 Jiangsu China; 4 University of Chinese Academy of Sciences, No.19(A) Yuquan Road, Beijing, 100049 China; 5 Department of Laboratory Animal Science, Fudan University, 130 Dong’an Road, Shanghai, 200032 China; 6 Department of Urology, Shanghai General Hospital, Shanghai Jiao Tong University School of Medicine, 100 Haining Road, Shanghai, 200080 China; 7 Department of Urology, Shanghai General Hospital of Nanjing Medical University, Shanghai, 200080 China

**Keywords:** Benign prostatic hyperplasia, *Rauwolfia vomitoria*, ER stress, Autophagy, Apoptosis

## Abstract

**Background:**

The current drug treatments for benign prostatic hyperplasia (BPH) have negative side effects. Therefore, it is important to find effective alternative therapies with significantly fewer side effects. Our previous study revealed that *Rauwolfia vomitoria* (RWF) root bark extract reversed BPH development in a rat model. However, the molecular mechanism of its inhibitory effects on BPH remains largely unknown.

**Methods:**

BPH-1 and WPMY-1 cell lines derived from BPH epithelial and prostatic stromal compartments were selected to investigate how RWF extract inhibits BPH *in vitro* by MTT and flow cytometry assays. Microarray, quantitative real-time PCR, immunoblotting, and GFP-LC3 immunofluorescence assays were performed to evaluate the effects of RWF extract on endoplasmic reticulum stress (ER stress) and autophagic apoptosis pathways in two cell lines. A human BPH *ex vivo* explant assay was also employed for validation.

**Results:**

RWF extract treatment decreased cell viability and induced apoptotic cell death in both BPH-1 and WPMY-1 cells in a concentration-dependent manner with the increase of pro-apoptotic PCDC4 protein. RWF extract induced autophagy by enhancing the levels of autophagic genes (*ULK2* and *SQSTM1/p62*) and the LC3II:LC3I ratio, with the increase of GFP-LC3 puncta. Moreover, RWF extract activated PERK- and ATF6-associated ER stress pathways by inducing the transcriptional levels of *EIF2AK3/PERK*, *DDIT3/CHOP* and *ATF6*, accompanied by the reduction of BiP protein level, but not its mRNA level. Another ER stress pathway was not induced by RWF extract, as manifested by the lack of XBP1 splicing. Pharmacological inhibition of autophagy by 3-methyladenine abrogated apoptosis but not ER stress; while inhibition of ER stress by 4-phenylbutyrate alleviated the induction of autophagy and apoptosis. In addition, pretreatments with either 3-methyladenine or 4-phenylbutyrate suppressed RWF extract-induced cytotoxicity. Notably, the inductions of PERK- and ATF6-related stress pathways and autophagic apoptosis were confirmed in a human BPH *ex vivo* explant.

**Conclusions:**

Our data have demonstrated that RWF extract significantly suppressed the viabilities of BPH epithelial cells and BPH myofibroblasts by inducing apoptosis via upregulating ER stress and autophagy. These data indicate that RWF extract is a potential novel alternative therapeutic approach for BPH.

**Supplementary Information:**

The online version contains supplementary material available at 10.1186/s12906-022-03610-4.

## Background

Benign prostatic hyperplasia (BPH) is a common age-related lesion of the prostate, manifested by the nonmalignant hyperproliferation of prostatic epithelial and stromal cells [1–3]. BPH is detected in more than 50% of men over 50 years old, while as high as 90% of men over the age of 80 have the symptoms of BPH. The marked enlargement of the prostate in men with BPH leads to lower urinary tract symptoms (LUTS) [3]. BPH will eventually cause urinary disorders, such as frequent and urgent urination, bringing physical and mental pain to patients and seriously affecting their quality of life. Although there are a number of drugs available to treat BPH, such as 5α-reductase inhibitors and α-blockers, their applications are limited due to intense side effects, such as vascular deficits, sexual dysfunction, hyperglycemia, and erectile dysfunction [4, 5]. In addition, the drugs must be used for a long time and cannot thoroughly cure BPH [6, 7]. There is an urgent need for alternative treatments for BPH with clear efficacy, fewer side effects, and lower cost.

Herbal extract with its milder effectiveness is deemed to be a complementary and alternative therapeutic method to BPH. Saw palmetto (Serenoa repens) extract is one of the most well-studied herbal extracts to treat BPH [8, 9]. Currently, a number of promising anti-BPH herbal extracts have been reported, including Pao Pereira and Stauntonia hexaphylla extracts [10, 11]. *Rauwolfia vomitoria* (RWF), a kind of valuable herbal plant, grows widely in the tropical zones of Africa and Asia. The RWF extract contains bioactive monoterpene and beta-carboline alkaloids, and is reported to treat many diseases, such as impaludism, gastrointestinal diseases, and cancer [12, 13]. Recently, we found that RWF extract could reverse the development of BPH by suppressing 5α-reductase and androgen receptor in a BPH rat model, similar to finasteride [14]. Interestingly, its side effects were milder than finasteride, with significantly lower toxicity to sperm development in comparison to finasteride [14]. Taken together, these data suggest that RWF extract may be a promising and relatively safe agent for BPH. However, the molecular mechanism underlying the anti-BPH effects of RWF extract needs to be further elucidated.

Endoplasmic reticulum (ER) stress is caused by structural or functional imbalance of the ER, due to the accumulation of unfolded or misfolded proteins in cells [15–17]. Protein kinase RNA-like ER kinase (PERK), inositol-requiring enzyme 1 (IRE1), and activating transcription factor 6 (ATF6) are ER-resident transmembrane proteins to sense ER stress. When unfolded or misfolded proteins bind to BiP/GRP78, the release and oligomerization of PERK are induced, and subsequent autophosphorylation of PERK triggers its activity to further phosphorylate eIF2α and increase the translation of ATF4. As a transcription factor, ATF4 can induce the expression of genes involved in autophagy, as well as cell death, such as C/EBP-homologous protein (CHOP). Hence, in response to the ER stress, basal autophagy can be activated to degrade the unfolded or misfolded proteins by lysosomes and maintain intracellular homeostasis [18, 19]. However, persistent or intense ER stress can lead to apoptosis [19].

In this study, we used two typical prostatic cell lines (BPH-1 and WPMY-1 cells), representing two major hyperproliferative cell types from BPH epithelial and stromal compartments, to investigate the effects of RWF extract on BPH. We examined the cytotoxic effects of RWF extract on these two cell lines, and performed gene expression profiling to identify the molecular events in ER stress and autophagic apoptosis induced by RWF extract. The results were further confirmed in ex vivo BPH explants treated with RWF extract.

## Materials and methods

### Chemical and reagents

The RWF extract (generously provided by Maison Beljanski, New York, NY) was prepared with aqueous-alcoholic extraction of the root bark from the tropical shrub *Rauwolfia vomitoria*, followed by a spray drying to produce a free-flowing powder form. The final product is standardized using HPLC for the active component alstonine, and reserpine is undetectable. Quality control includes tests for pesticides, heavy metals, bacteria, yeast, and mold. For experiments, the RWF extract was dissolved in DMSO and further diluted with sterile phosphate buffered saline (PBS) to 50 mg/ml stock solutions. The RWF extract was stored in aliquots at -80 °C until use. Cisplatin (Cat #: HY-17394), ER inhibitor 4-phenylbutyrate (4-PBA; Cat #: HY-A0281), and autophagy inhibitor 3-methyladenine (3-MA; Cat #: HY-19312) were purchased from MedChemExpress (MCE) Co., Ltd (Shanghai, China).

### Cell culture

Human BPH-1 cell line, derived from BPH tissues, was a gift obtained from Dr. Simon Hayward (Vanderbilt University Medical Center, Nashville, TN), while human prostate myofibroblast WPMY-1 cells were purchased from CBTCCCAS (The Cell Bank of Type Culture Collection of Chinese Academy of Sciences, Shanghai, China). BPH-1 and WPMY-1 cell lines were cultured as monolayers in RPMI 1640 and DMEM respectively, supplemented with 10% fetal bovine serum (FBS; Life Technologies, Carlsbad, CA) and penicillin (100 U/ml) plus streptomycin (100 μg/ml) (S110JV, BasalMedia, Shanghai, China) at 37 °C and 5% CO_2_.

### Cytotoxicity assay

BPH-1 cells (2 × 10^3^ cells/well) and WPMY-1 cells (6 × 10^3^ cells/well) were seeded in 96-well plates, respectively. On the 2^nd^ day, cells were treated with 0, 125, 250, and 500 μg/ml RWF extract for 0, 24, 48, and 72 h. Cell viability was measured by staining cells with 10 μl of 5 mg/ml MTT (Cat #: 143315, Biofroxx) to each well in a 37℃ incubator for 4 h, followed by dissolution with 100 μl DMSO for each well. Optical density (OD) was measured by a microplate reader (SpectraMax M5, Molecular Devices, Sunnyvale, CA) at 490 and 680 nm. For the rescue assay, cells were pre-treated with an ER stress inhibitor 4-PBA (250 µM) or an autophagy inhibitor 3-MA (500 µM) for 2 h, followed by the addition of RWF extract (500 µg/ml).

### Flow cytometry analysis

Cell apoptotic rates were examined by FITC Annexin V Apoptosis Detection Kit I (Cat #556547, BD Biosciences, Franklin Lakes, NJ), according to the manufacturer’s instructions. Briefly, BPH-1 and WPMY-1 cells were cultured in RPMI1640 and DMEM (containing 10% FBS) in 6-well plates at densities of 50,000 cells/well and 100,000 cells/well, respectively. The cells were then treated with various concentrations of RWF extract (0, 125, 250, and 500 μg/ml) for 72 h. The 72 h treatment of cisplatin (5 μM) served as the positive control. Both the floating and adherent cells were harvested and washed with PBS. Afterwards, cells were stained with both Annexin V-FITC and propidium iodide (PI) in a dark place for 15 min. The cell death patterns were examined by a flow cytometer with 10,000 events and analyzed by FlowJo software (ver10.0.7r2, Ashland, OR).

### Immunoblotting assay

Cells were lysed using a modified RIPA Lysis Buffer (150 mM NaCl, 1 mM Na_3_VO_4_, 50 mM Tris–HCl-pH 7.5, 25 mM NaF, 1% NP-40, 0.5% sodium deoxycholate, 0.1% SDS and 1% phosphatase inhibitor cocktails) on ice for 30 min. After lysates were centrifuged at 12,000 g to remove debris, protein concentrations were measured by BCA Protein Assay Kit (Cat #: P0011, Beyotime, Shanghai, China). 10–25 μg proteins were fractionated by SDS-PAGE, followed by the transfer to nitrocellulose membrane (Millipore, Billerica, MA). The membranes were then cropped based on the prestained protein marker for the subsequent incubations of corresponding primary antibodies (Supplementary Table 1) at 4 ℃ for 12 h. The incubation of secondary antibody was performed at room temperature for 1 h after TBST solution washing. Blots were visualized by adding SuperSignal™ West Femto Maximum Sensitivity Substrate (Thermo Fisher Scientific, Waltham, MA) in the Mini Chemiluminescent Imaging and Analysis System (Beijing Sage Creation Science, Beijing, China).

### Gene expression profiling

Total RNA was collected by TRIzol reagent (Thermo Fisher Scientific) from BPH-1 and WPMY-1 cells exposed to the 250 μg/ml of RWF extract for 24 h. Gene expression profiling analyses were performed by Shanghai Baygene Biotechnologies (Shanghai, China) using the Affymetrix gene chips (Human Transcriptome Array 2.0). The microarray data were deposited in the NCBI GEO Database with the accession number: GSE151643.

### Quantitative reverse-transcription polymerase chain reaction (qRT-PCR)

Total RNA from BPH cells or human BPH tissues treated with the indicated concentration of RWF extract was extracted by TRIzol. Afterwards, 1 μg of total RNA was used for cDNA synthesis using a Hifair® II 1st strand cDNA Synthesis SuperMix for qPCR (Cat #: 11123ES60, YEASEN, Shanghai, China). qRT-PCR was performed in triplicates using designated gene-specific primers (Supplementary Table 2) by CFX96 Touch Real-Time PCR Detection System (Bio-Rad, Hercules, CA). The expression of *ACTB* gene was used as an internal control.

### XBP1 splicing assay

The total RNA and cDNA from BPH-1 and WPMY-1cells were prepared as described above. The splicing variants of XBP1 were amplified using specific primers (Supplementary Table 3) by RT-PCR. The PCR products were then separated on 2.0% agarose gel and visualized by the staining with Ultra GelRed dye (Cat #: GR501-01, Vazyme Biotech, Nanjing, China).

### GFP-LC3 analysis

GFP-LC3 lentiviral particles were produced by co-transfection of with GPF-LC3 plasmid together with psPAX2 packaging plasmid and pMD2.G envelope plasmid to 293 T cells using the transfection reagent polyethyleneimine (PEI; Cat #: 408727, Sigma-Aldrich, St. Louis, IL). 48 h after transfection, lentivirus-containing supernatant was collected and filtered using Millex-HV syringe filter unit, low adsorption 0.45 μM (Cat #: SLHU033RB, MERCK, Burlington, MA). BPH-1 cells were infected three times and WPMY-1 cells were infected twice with the virus-containing media in the presence of 8 μg/ml polybrene (Cat #: TR-1003, Sigma-Aldrich). Puromycin (Cat #: 58–58-2, Selleck, Houston, TX) was used to select infected cells (2 μg/ml for WPMY-1 and 15 μg/ml for BPH-1 cells). GFP-LC3 stably transduced BPH-1 and WPMY-1 cells were treated with/without RWF (250 μg/ml and 500 μg/ml), respectively. After 24 h growing on cover glasses, cells were washed in PBS, followed by the fixation in 4% paraformaldehyde before the visualization by LEICA SP8 confocal microscope. The number of GFP-LC3 puncta per cell was counted in 30 individual cells for each group. Brown-Forsythe and Welch ANOVA test was performed to determine the statistical significance of differences among multiple groups.

### Human BPH ex vivo assay

Human BPH ex vivo explants (*n* = 6) were obtained from BPH patients with radical prostatectomy at Shanghai General Hospital, Shanghai Jiao Tong University (China). Briefly, fresh tissues were immersed in sterile DMEM/F-12 medium (Cat #: 10–920-CV, Corning, New York, NY) on ice and transported to the laboratory immediately. After the burnt part of the fresh tissue was removed, BPH tissue was dissected into 3–5 mm^3^ pieces. The BPH explants were cultured on the sterile gelatin sponge (Cat #: HSD-B, Hushida Medical Care Technology, Nanchang, China), which was pre-soaked in DMEM-F12 medium containing 10% FBS, 0.01 mg/ml hydrocortisone (Cat #: H0135, Sigma-Aldrich), antibiotic/antimycotic solution (Cat #: S120JV, BasalMedia), and 0.01 mg/ml insulin (Cat #: I1882, Sigma-Aldrich) in 6-well-plates at 37 °C, as described previously [20–22]. The next day, tissues were treated with various concentrations of RWF extract (0, 250, and 500 μg/ml) in fresh medium for 24 h (to induce ER stress and autophagy) or 48 h (to induce apoptosis).

### Statistical analysis

The experiments were performed in three independent replicates, and the statistical analyses were performed using Student’s *t* test in GraphPad Prism 7.0 software (GraphPad, San Diego, CA).ns, not significant, * *p* < 0.05, ** *p *< 0.01, *** *p* < 0.001.

## Results

### RWF extract reduced cell survival in BPH-1 and WPMY-1 cells

**Fig. 1:**
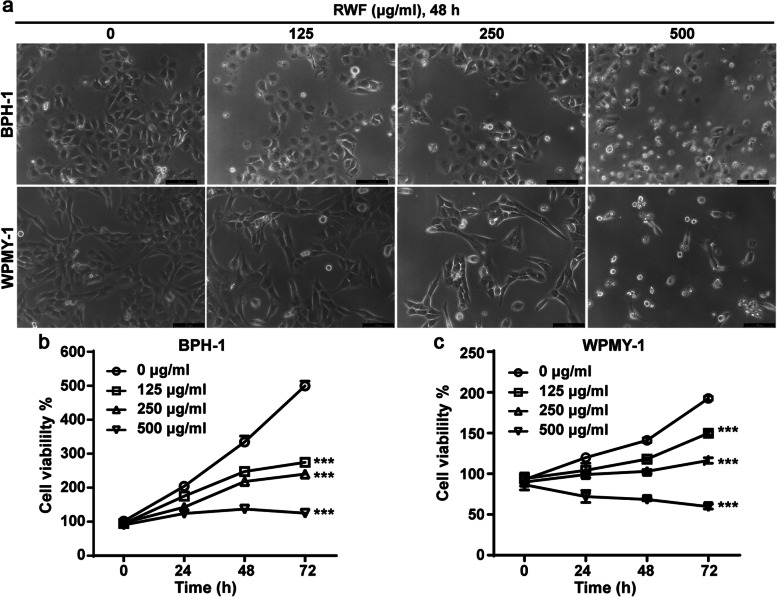
RWF extract inhibited proliferation in BPH-1 and WPMY-1 cells. **a** Morphological changes of BPH-1 and WPMY-1 cells under the treatment of RWF extract for 48 h. Scale bar, 100 μm. **b**, **c** The cytotoxic effects of RWF extract on cell viability by the MTT assay. RWF extract was used to treat BPH-1 and WPMY-1 cells at the indicated concentrations for 0, 24, 48, and 72 h. Data were presented as mean ± SD; *** *p* < 0.001 indicated a significant difference between cells treated with RWF extract at indicated concentrations and non-treated cells

BPH is characterized by non-malignant uncontrolled proliferation of the epithelial and stromal portions in prostatic glands [1, 2]. To investigate whether RWF exerts any inhibitory effects on BPH in vitro, we enrolled two BPH cell lines, BPH-1 cell line (derived from BPH epithelial cells) and WPMY-1 myofibroblast line (derived from prostate stroma). Both cell lines were exposed to the indicated concentration of RWF extract for 48 h, followed by MTT assay, respectively. We observed more floating cells and fewer adherent cells in the group treated with a higher concentration of RWF extract than in the groups treated with a lower concentration (Fig. 1a). 500 μg/ml of RWF extract induced the maximal proportion of dying cells in both BPH-1 and WPMY-1 cells. In line with this finding, the cytotoxicity assay showed that compared with the vehicle group, RWF extract effectively and significantly inhibited cell survival of both cell lines in a concentration- and time-dependent manner (Fig. 1b, c).

### RWF extract induced apoptosis in BPH-1 and WPMY-1 cells in a dose-dependent manner

**Fig. 2:**
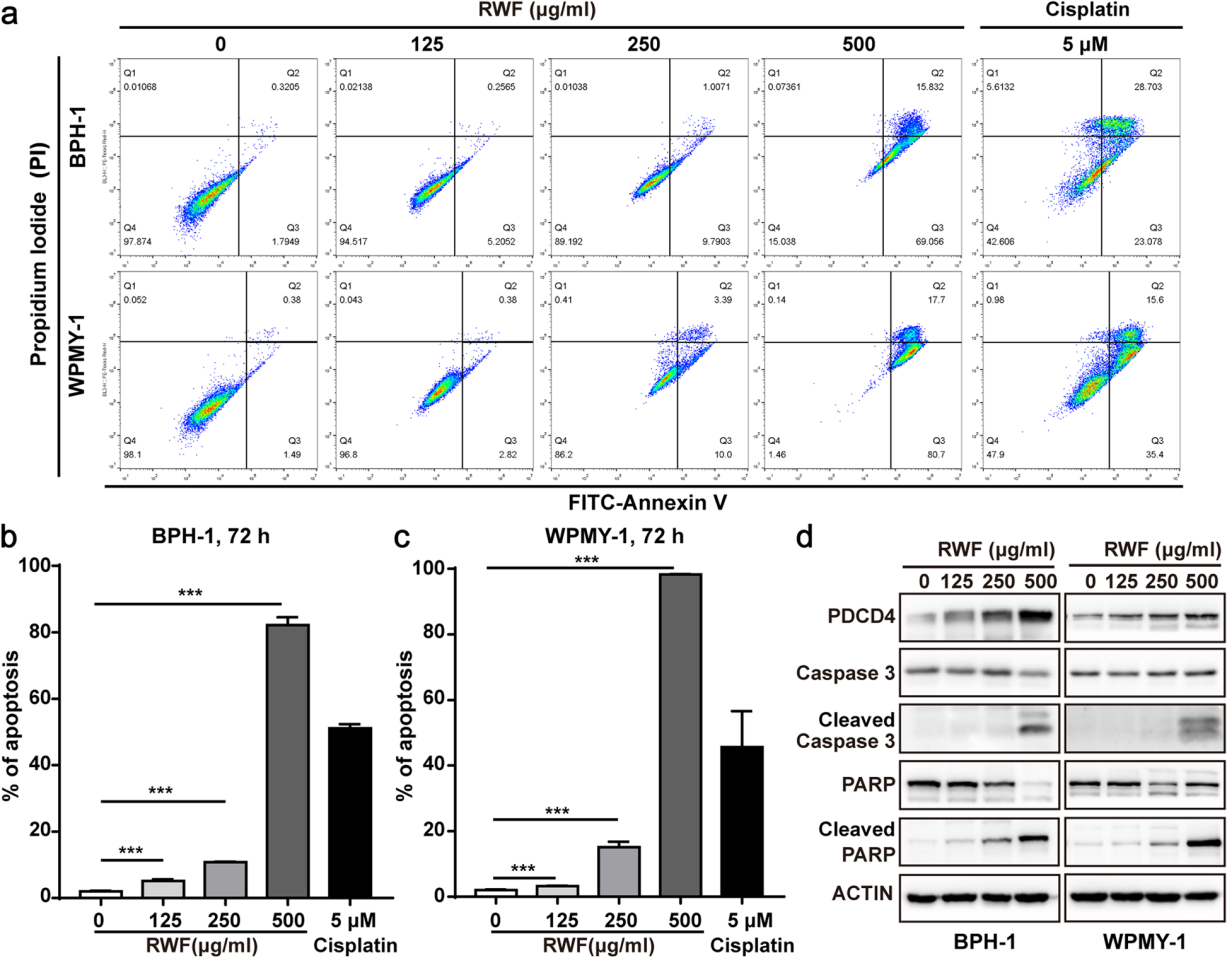
RWF extract induced cell apoptosis of BPH-1 and WPMY-1 in a concentration-dependent manner**. a** BPH-1 and WPMY-1 cells were treated with the indicated concentration RWF extract for 72 h, followed by staining with FITC-Annexin and Propidium Iodide (PI). The cells treated with 5 μM cisplatin for 72 h were used as the positive controls. **b**,** c** Apoptotic ratio was determined by flow cytometry. Data were presented as mean ± SD; *** *p* < 0.001. **d** The expression levels of pro-apoptosis-related proteins in the presence of RWF extract by immunoblotting analysis in BPH-1 and WPMY-1 cells

To further investigate whether the cytotoxicity induced by RWF extract is due to the increase in apoptosis, we treated both BPH-1 and WPMY-1 cells with 0, 125, 250, and 500 μg/ml of RWF extract for 72 h, followed by Annexin V/PI double staining assay. Flow cytometry data showed that RWF extract induced apoptosis in a concentration-dependent manner, while 500 μg/ml of RWF extract strikingly induced 82.20 ± 1.44% and 98.26 ± 0.07% apoptotic cell population in BPH-1 and WPMY-1 cells, respectively. As a positive control, cisplatin induced 51.11 ± 0.73% and 45.65 ± 6.33% in BPH-1 and WPMY-1 cells for 72 h, respectively (Fig. 2a-c). To further corroborate the flow cytometry data, we carried out the immunoblotting assay. As shown in Fig. 2d, RWF extract increased the cleavages of Caspase 3 and its substrate PARP in both cell lines. Since PDCD4, a pro-apoptotic protein, is sensitive to herbal extract-induced apoptosis in BPH-1 and WPMY-1 cells [20], we assessed its expression level and found it was increased by RWF extract treatment in a concentration-dependent manner. These data showed that RWF extract induced apoptosis in both BPH-1 and WPMY-1 cells.

### RWF extract induced autophagic gene expression in BPH-1 and WPMY-1 cells

**Fig. 3:**
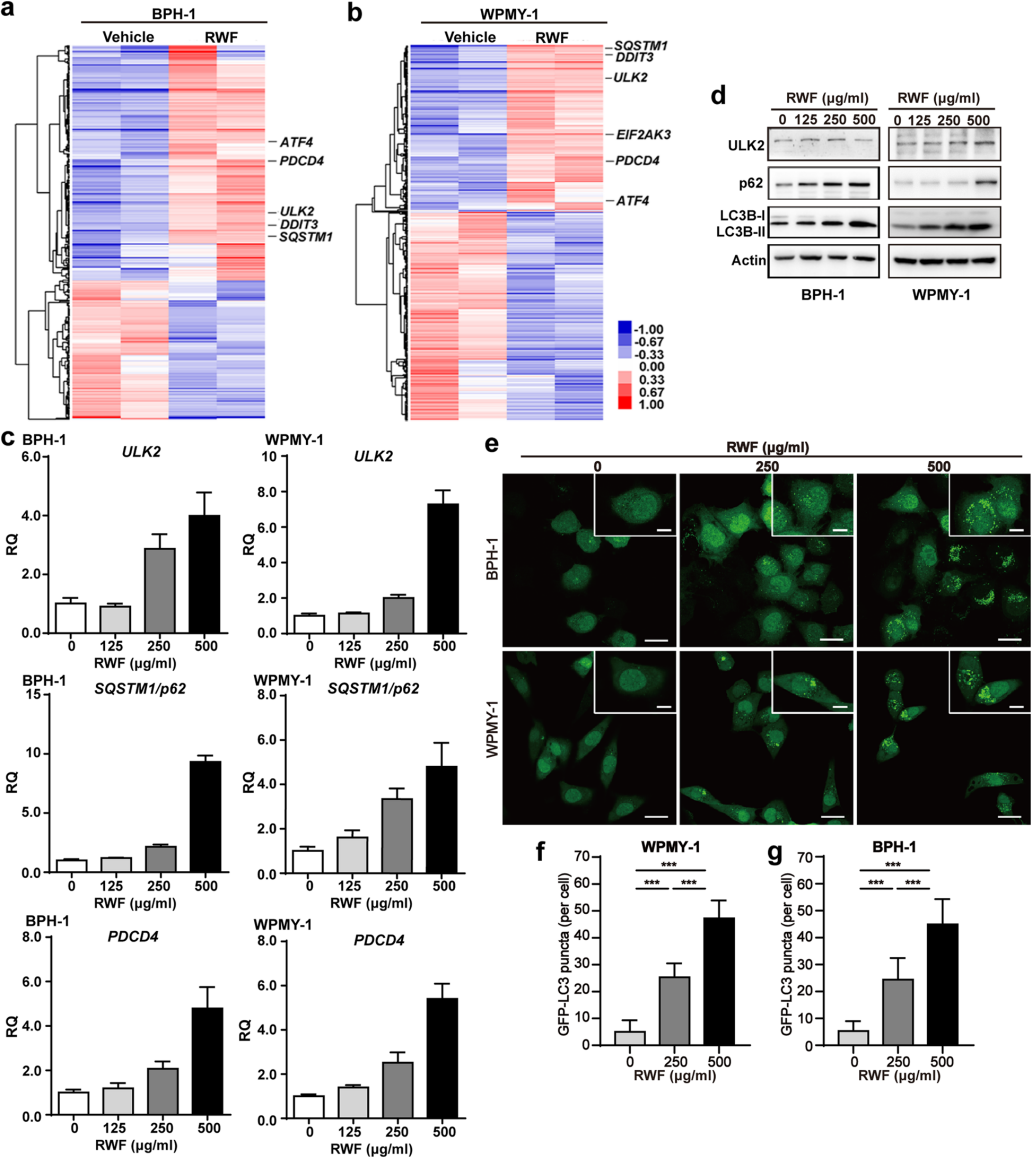
RWF extract regulated autophagy pathway in BPH-1 and WPMY-1 cells. **a**, **b** The expression profiling analysis of BPH-1 and WPMY-1 cells exposed to RWF extract (250 μg/ml) or vehicle for 24 h by microarray. The expression levels of autophagy-related genes (*ULK2* and *SQSTM1/p62*) and ER-stress-associated gene (*EIF2AK3/PERK, ATF4*, and *DDIT3/CHOP*) were indicated. **c**,** d** The mRNA and protein levels of autophagy-associated genes in BPH-1 and WPMY-1 cells treated with RWF extract for 24 h were detected by qRT-PCR (**c**) and immunoblotting (**d**) assays, respectively. **e** BPH-1 and WPMY-1 cells with the stable expression of GFP-LC3 were treated with RWF (250 or 500 μg/ml) or vehicle for 24 h. GFP-LC3 puncta patterns were examined under a LEICA confocal microscope. Scale bars: 25 μm; inset, 10 μm. **f**,** g** Quantification of the number of LC3-GFP puncta per cell in BPH-1 (**f**) and WPMY-1 cells (**g**). 30 individual cells were counted in each group. ***p < 0.001

Next, to explore how RWF extract induced cytotoxic effects on BPH-1 and WPMY-1 cells, gene expression profiling was performed using the Affymetrix HTA2.0 microarray chips. Using 1.3-fold as the cutoff value, we observed the upregulation of 234 coding genes and 138 non-coding transcripts, as well as the downregulation of 125 coding genes and 96 non-coding transcripts in RWF extract-treated BPH-1 cells, compared to the vehicle-treated BPH-1 cells (Fig. 3a). In WPMY-1 cells, RWF extract treatment upregulated 678 coding genes and 510 non-coding transcripts, as well as the downregulation of 810 coding genes and 679 non-coding transcripts (Fig. 3b). When comparing the upregulated genes in two cell lines, we found a group of genes involved in cell death were induced in both cells by RWF extract. In addition to a pro-apoptotic gene (*PDCD4*), which was shown to be upregulated at the protein level (Fig. 2d), two macro-autophagy associated genes (*ULK2* and *SQSTM1/p62*) were also induced, suggesting RWF extract could also activate the autophagic pathway.

We then validated that RWF extract induced the transcriptional levels of *ULK2*, *SQSTM1/p62*, and *PDCD4* in a concentration-dependent manner (Fig. 3c). To further address whether RWF extract induced autophagy, we performed the immunoblotting assay and found that, in addition to the induction of ULK2 and p62 at protein levels, RWF extract also increased the ratio of LC3II/LC3I proteins, an autophagy marker (Fig. 3d). Since LC3 can form puncta when autophagy is activated, we generated BPH-1 and WPMY-1 cells with the stable expression of exogenous GFP-LC3 by lentiviral transduction. In these GFP-LC3-labelled BPH-1 and WPMY-1 cells, administration of RWF extract strikingly caused more GFP-LC3 puncta in both cells (Fig. 3e-g). Interestingly, more GFP-LC3 puncta were found in the group with 500 μg/ml RWF extract than those with 250 μg/ml RWF extract. Altogether, these data indicated that RWF extract stimulated autophagy in both BPH-1 and WPMY-1 cells in a concentration-dependent manner.

### RWF extract induced ER-stress pathway in BPH-1 and WPMY-1 cells

**Fig. 4:**
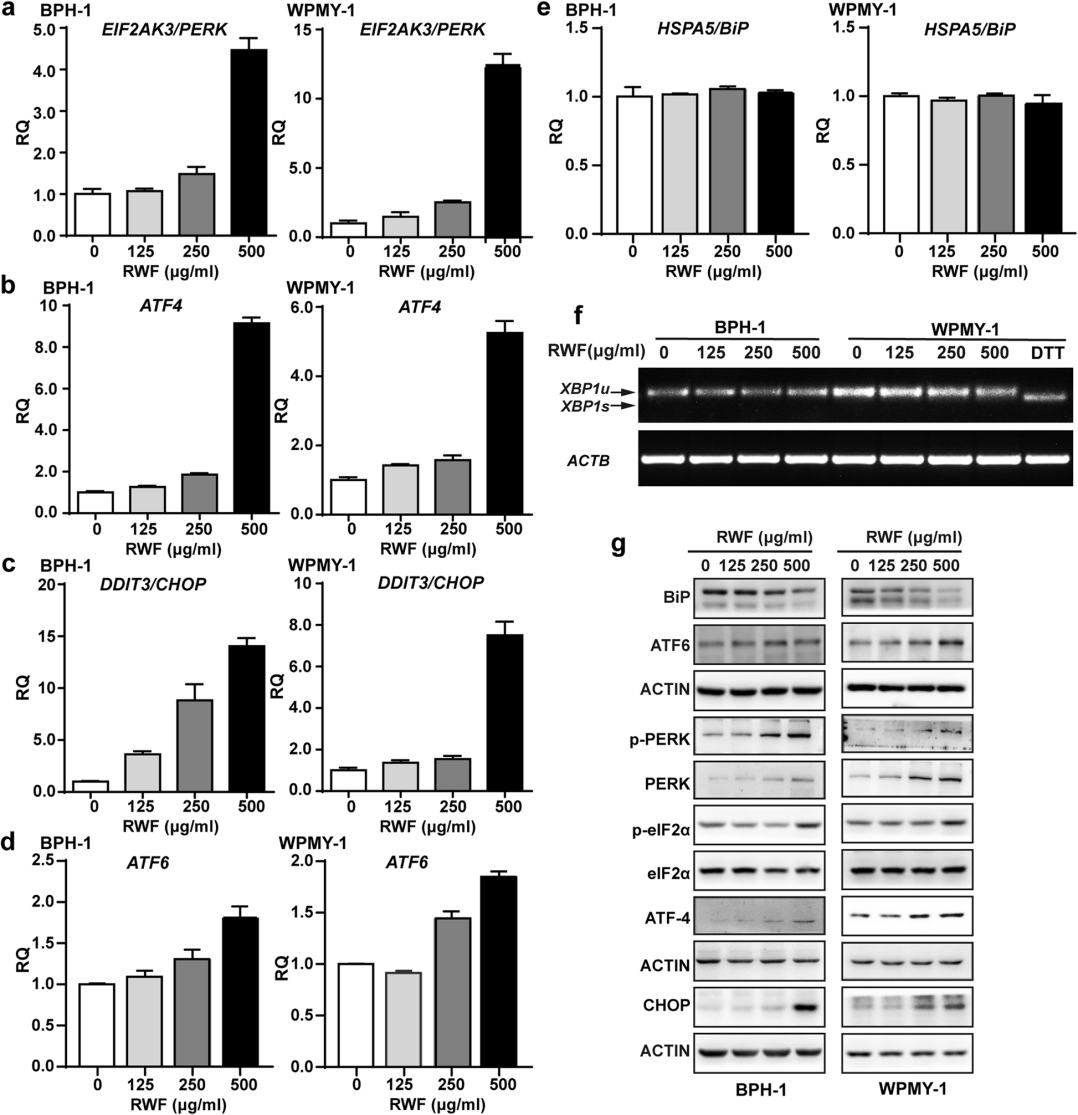
RWF extract induced ER-stress pathways in BPH-1 and WPMY-1 cells. **a-e** The mRNA expression levels of ER stress-associated genes in BPH-1 and WPMY-1 cells by qRT-PCR. **f** BPH-1 and WPMY-1 cells were treated with 0, 125, 250 and 500 μg/ml RWF for 24 h. The cells treated with 10 mM DTT for 4 h were used as the positive controls for XBP1 gene splicing. The amplicon sizes of XBP1u and XBP1s were 261 bp and 235 bp, respectively. u, unspliced form; s, spliced form. **g** Immunoblots of ER stress-associated proteins in BPH-1 and WPMY-1 cells treated with RWF extract for 24 h

The unfolded protein response (UPR) of eukaryotic cells maximizes protein folding fidelity and maintains ER function, which is regulated by three sentinel sites: PKR-like ER Kinase (PERK), activating transcription factor 6 (ATF6), and inositol-requiring protein 1α (IRE1α). Upon induction of ER stress, BiP/GRP78 is recruited by misfolded proteins, leading to its dissociation with PERK, IRE1, and ATF6 proteins. Such releases eventually activate PERK, IRE1, and ATF6 pathways, and finally, induce the UPR signaling cascade [18]. Interestingly, in the aforementioned gene expression profiling, we also observed three key genes (*EIF2AK3/PERK*, *ATF4*, and *DDIT3/CHOP*) were induced in both cell lines. Hence, we first confirmed that these three ER stress-associated genes were increased at the mRNA level by RWF extract in a concentration-dependent manner (Fig. 4a-c). Furthermore, we examined whether the expression levels of BiP and the other two signaling cascades in UPR pathways were altered or not. By qRT-PCR assay, we found that HSPA5/BiP mRNA expression was not altered by RWF extract treatment, whereas ATF6 mRNA level was up-regulated (Fig. 4d, e). Interestingly, RT-PCR data showed that XBP-1 was not cleaved after RWF extract treatment, indicating that RWF extract did not activate the IRE1α pathway (Fig. 4f). Next, we performed the immunoblotting assay and confirmed that PERK, ATF4, CHOP, and ATF6 proteins were all induced by RWF extract treatment, while BiP displayed a concentration-dependent decrease under the treatment of RWF extract (Fig. 4g). In addition, the phosphorylation levels of PERK(T980) and eIF2α(S51) also increased in response to RWF extract treatment, indicating the activation of PERK signaling pathway in BPH-1 and WPMY-1 cells (Fig. 4g). Taken together, these data indicated that RWF extract reduced BiP expression and activated PERK- and ATF6-associated ER stress pathways, resulting in UPR signaling activation in both BPH epithelial and stromal cells.

### Abrogation of ER stress reduced RWF extract-induced autophagic apoptosis

The exact molecular mechanism underlying RWF extract-induced cell death has not been fully elucidated, particularly in hyperplastic nonmalignant cells. Though we identified the induction of autophagy and ER stress by RWF extract, both cellular processes play promoting or inhibitory roles in cell survival, which are dependent on cell context [23, 24]. In addition, crosstalk between autophagy and ER stress has also been identified [25, 26]. Hence, it is important to characterize how RWF extract induced cell death of BPH-1 and WPMY-1 cells.

**Fig. 5:**
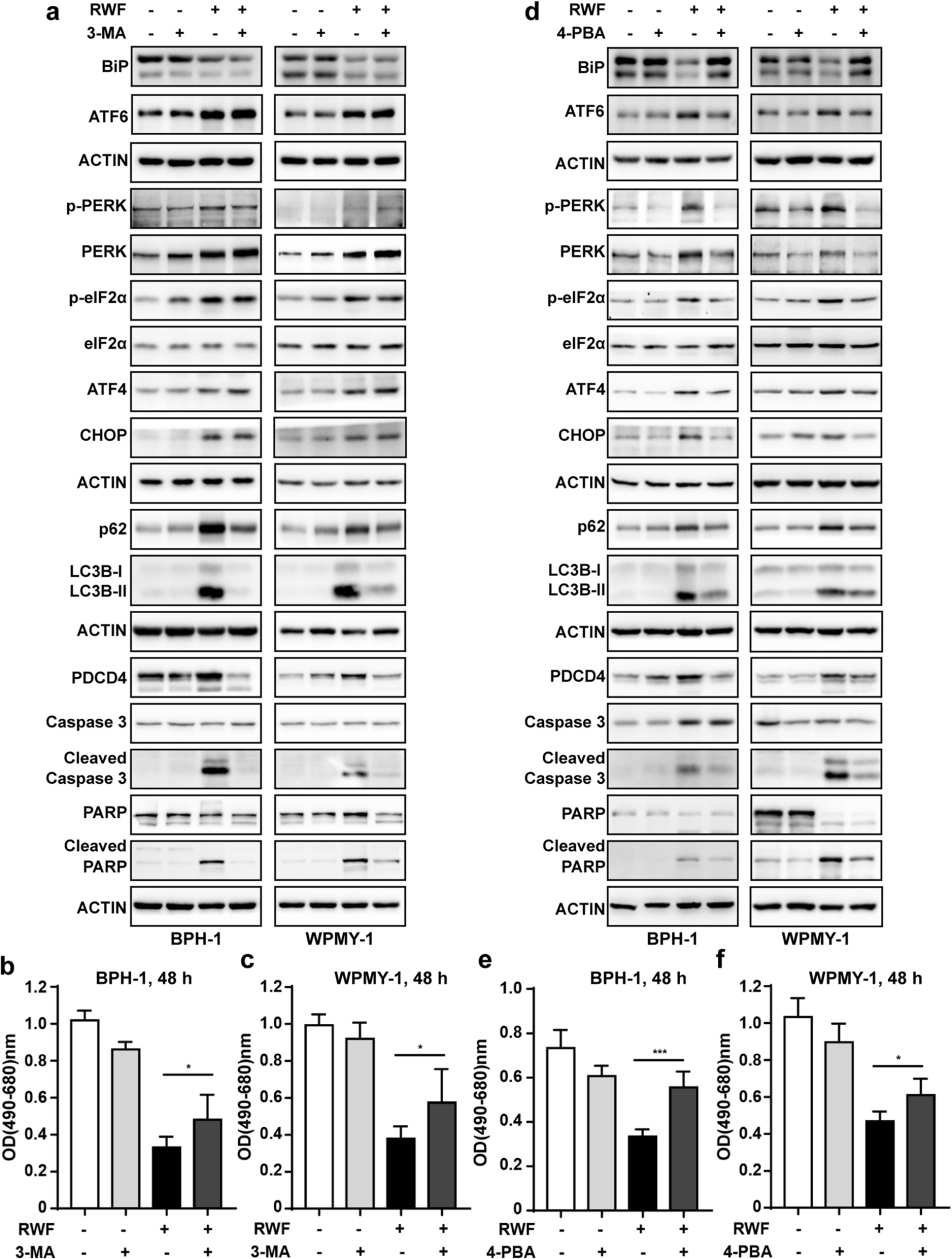
RWF extract induced apoptosis of BPH-1 and WPMY-1 cells via activating the ER and autophagy pathways. **a** The ER stress, autophagy and apoptosis-associated proteins were analyzed by immunoblot analysis. BPH-1 and WPMY-1 cells were pretreated with or without 500 µM autophagy inhibitor 3-MA for 2 h in advance and treated with or without 500 µg/ml RWF extract for another 24 h (to induce ER stress and autophagy) or 48 h (to induce apoptosis). **b**, **c** Cell viability was assessed by MTT assay in BPH-1 and WPMY-1 cells. **d** BPH-1 and WPMY-1 cells were pre-treated with or without 250 µM ER-stress inhibitor 4-PBA for 2 h in advance and treated with or without 500 µg/ml RWF extract for another 24 h (to induce ER stress and autophagy) or 48 h (to induce apoptosis). **e**, **f** Cell viability was assessed by MTT assay in BPH-1 and WPMY-1 cells. Data were presented as mean ± SD of at least three independent experiments. *p < 0.05, or ***p < 0.001

As a widely used inhibitor of autophagy via its inhibitory effect on class III PI3K, 3-MA was chosen to pretreat cells to block autophagy [27]. As shown in Fig. 5a, the inhibition of autophagy by 3-MA strikingly abrogated the induction of pro-apoptotic protein PDCD4 and the cleavages of Caspase 3 and PARP by RWF extract. However, RWF extract-induced expression changes of ER stress-associate proteins (BiP, ATF6, PERK, ATF4, and CHOP) and phosphorylation levels of PERK and eIF2α were not observed in the presence of 3-MA (Fig. 5a). Notably, the inhibition of autophagy by 3-MA partially reversed the RWF extract-induced cytotoxicity in both BPH-1 and WPMY-1 cells (Fig. 5b, c). These data clearly indicated that RWF extract induced autophagic apoptosis.

Since autophagy inhibition does not affect RWF extract-induced ER stress, we hypothesized that RWF may activate ER stress to eventually induce autophagic apoptosis. To confirm this hypothesis, we treated the cells with 4-PBA, an ER stress inhibitor, before treating the cells with RWF extract. 4-PBA successfully inhibited the changes of BiP, PERK, ATF4, CHOP, and ATF6 expression levels and activation of ER stress pathway caused by RWF extract (Fig. 5d). Of note, our data showed a prominent attenuation of RWF-induced autophagy and pro-apoptotic proteins in BPH-1 and WPMY-1 cells pretreated with 4-PBA. Moreover, suppression of ER stress pathway also abrogated the RWF extract-induced cell death (Fig. 5e, f). Hence, these data showed that RWF extract promoted cell apoptosis by activating the ER stress response and subsequent downstream autophagy and apoptotic pathways.

### RWF extract induced ER-stress and autophagic apoptosis in human BPH tissues

**Fig. 6:**
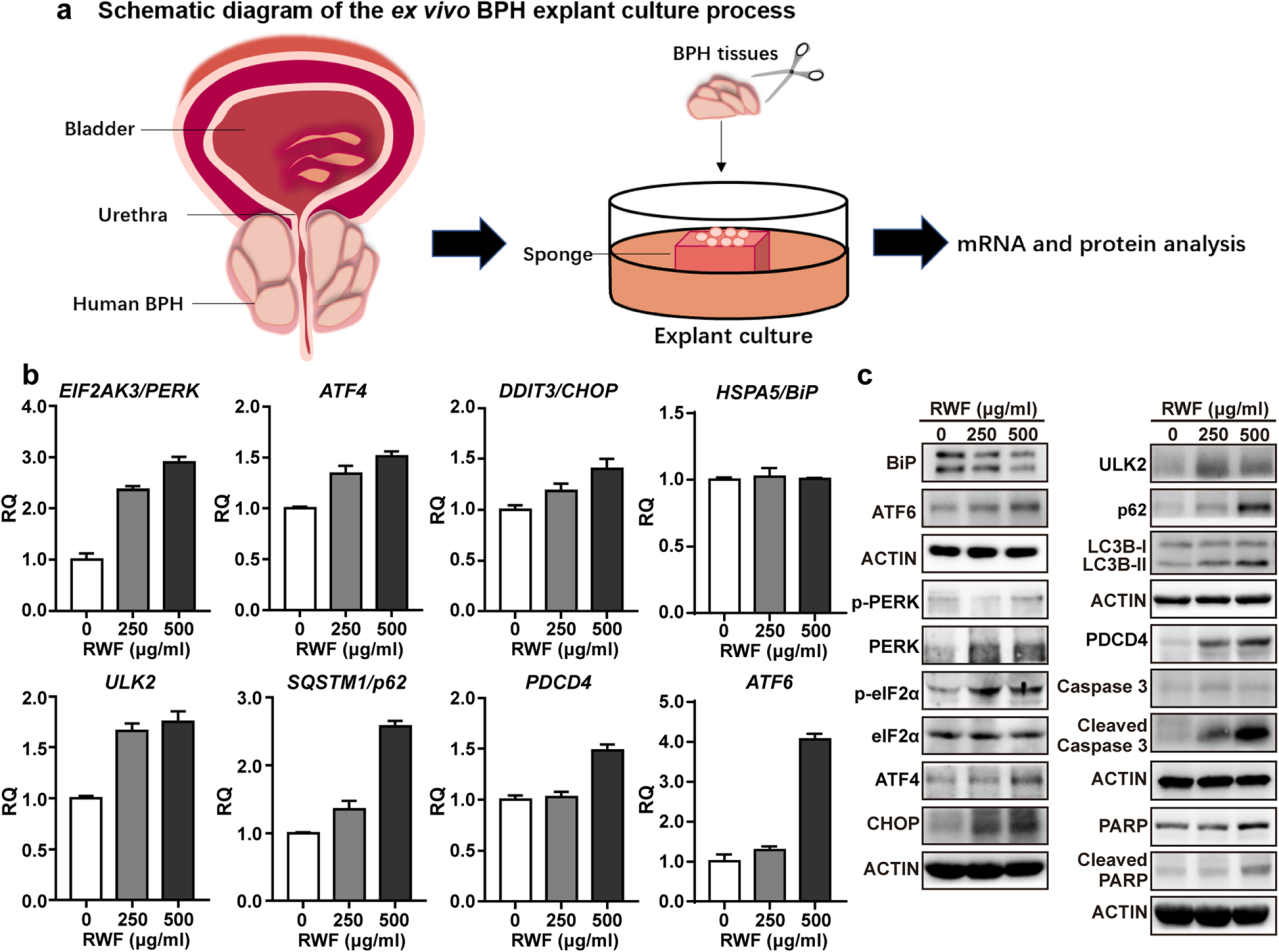
RWF extract inhibited ER stress, autophagy and apoptosis signaling pathways in human BPH tissues. **a** Schematic diagram of culturing BPH tissue *ex vivo* from human samples. BPH tissues were treated with 250 and 500 μg/ml RWF extract for 48 h. **b** The mRNA expression levels of ER-stress and autophagy-associated genes were measured by qRT-PCR in human BPH explants treated with 250and 500 μg/ml RWF extract for 24 h. Data were presented as mean ± SD. (*n* = 3). **c** The expression levels of target proteins from ER stress signaling pathway, pro-apoptotic and autophagy-related proteins were increased in a concentration-dependent manner in human BPH explants under the treatment of 250 and 500 μg/ml RWF extract for 24 h (to induce ER stress and autophagy) and 48 h (to induce apoptosis)

To further confirm RWF extract plays a significant role to suppress viability via targeting ER stress and autophagy signaling, human BPH tissues were cultured *ex vivo* and treated with the indicated concentrations of RWF extract (Fig. 6a). Consistent with previous *in vitro* results in BPH-1 and WPMY-1 cells, we found that RWF extract also induced ER stress and autophagy signaling pathway-related genes in human BPH tissues, without changing the mRNA levels of *HSAP5/BiP* (Fig. 6b). Moreover, consistent with the aforementioned immunoblotting data in BPH-1 and WPMY-1 cells, RWF extract reduced BiP protein levels, but increased the levels of other ER-stress-related proteins, as well as the autophagy and pro-apoptotic proteins in human BPH tissues (Fig. 6c).

## Discussion

**Fig. 7:**
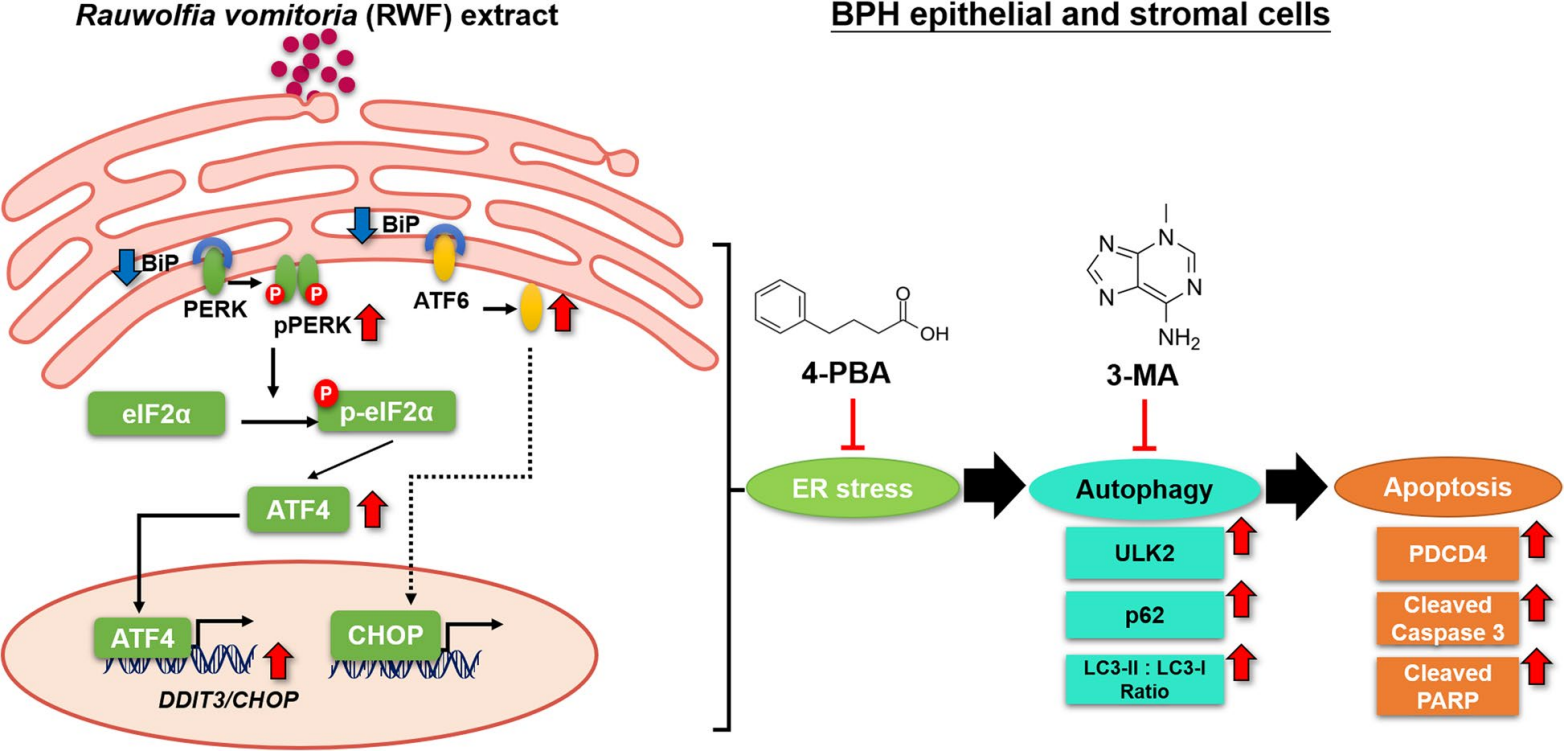
Schematic overview of mechanism how RWF extract inhibits BPH development. RWF extract induces the expression of BiP, ATF6, PERK, ATF4, and CHOP, and activates PERK- and ATF6-associated ER stress pathways, leading to autophagy and apoptosis

We previously reported the inhibitory effects of RWF extract on BPH development in a BPH rat model, with milder side effects compared with finasteride [14]. Here, we further dissected the molecular mechanism and found that RWF extract suppressed the viabilities of BPH epithelial and stromal cells in a concentration-dependent manner, through the induction of PERK/eIF2α/ATF4/CHOP signaling axis of ER stress that triggers autophagic apoptosis (Fig. 7). The inhibition of ER stress and autophagy reversed the RWF extract-induced cytotoxicity in both BPH-1 and WPMY-1 cells, respectively. Notably, the inductions of PERK- and ATF6-associated ER stress, autophagy, and apoptosis were confirmed in a human BPH *ex vivo* model.

Autophagy is an evolutionally conserved cellular stress response to control the quality of proteins and organelles, which acts as a pro-survival mechanism in general [23]. During BPH pathogenesis, the dysregulation of autophagy was reported to be associated with the inflammation status inside prostatic tissues [28]. In addition to the hyperplasic proliferation of both prostatic epithelial and stromal cells in BPH tissues, hypoxia and innervation of cholinergic pelvic nerve in the prostate tissues can also induce the activation of autophagy during BPH development [29–32]. However, autophagy possesses cytotoxic function besides the cytoprotective effect, which is highly context-dependent. Whether cytotoxic or cytoprotective autophagy is active appears to be associated with the duration and extent of autophagy [33]. Indeed, one of the therapeutic approaches for BPH patients is to increase the duration and extent of autophagy. For instance, as a 5α-reductase inhibitor, finasteride could significantly increase the expression of the autophagy-related protein LC3-II in human BPH tissues [34]. The autophagy in prostate epithelial cells could be induced by the reduced secretion of IGF-1 from prostatic stromal cells after the treatment with finasteride [35]. Several herbal extracts, including Stauntonia hexaphylla and Qianliexin capsule, a standardized traditional Chinese herbal preparation, have been reported to induce autophagy and apoptosis in BPH *in vivo* [11, 36]. In this study, we found that RWF extract could induce autophagic genes, *ULK2* and *SQSTM1/p62* at the transcriptional level in both BPH-1 and WPMY-1 cells. The increase of LC3II/LC3I ratio and puncta formation of LC3 inside cells further confirmed the induction of autophagy by RWF extract. Interestingly, during our manuscript preparation, an independent group reported that RWF extract suppressed autophagy and induced apoptosis in colorectal cancer cells [37]. Such discrepancy in the molecular mechanisms might be due to the difference between our non-malignant cell and cancer cell models. In our cell lines, we demonstrated that such induction of autophagy was essential for subsequent RWF extract-induced cell death. Hence, RWF extract induced autophagic apoptosis in both BPH epithelial and stromal cells.

ER stress functions as one of the upstream signals for autophagy [25]. Similar to autophagy, ER stress is one of the multiple-step processes that maintain intracellular homeostasis. The accumulation of unfolded and/or misfolded proteins can trigger the UPR and subsequent cytoprotective autophagy in order to recycle the damaged organelles and proteins. As one of three major signals to initiate UPR, PERK release from BiP protein induces its auto-phosphorylation and subsequent phosphorylation of eIF2α, which eventually activates eIF2α/ATF4/CHOP signaling pathway. The key autophagic gene p62 is a direct downstream target of the eIF2α/ATF4/CHOP signaling pathway [38]. Once the stress exceeds the threshold or goes on too long, UPR will eventually induce cell death. In response to the hyper-activation of PERK or ATF6, CHOP is one of the key downstream targets and its induction further activates a number of pro-apoptotic proteins to initiate autophagy and apoptosis [39]. Though the link between ER stress and BPH pathogenesis remains unclear, the treatment of 5α-reductase inhibitor finasteride significantly induced ER stress and apoptosis of spermatogenic cells in rat testis tissue [40]. Our study revealed that RWF extract induced the expression changes of key components in PERK- and ATF6-associated ER stress signalings, as well as the autophagic and apoptotic genes in both BPH-1 and WPMY-1 cells by unbiased microarray assays. The inhibition of ER stress by 4-PBA reversed the induction of autophagy and apoptosis by RWF extract, while the treatment with autophagy inhibitor, 3-MA, could only abrogate RWF extract-induced apoptosis. These data reinforced the notion that RWF extract induced ER stress and further activated autophagic apoptosis in BPH cells.

RWF extract has been reported to inhibit hyperproliferation of malignant cells, including prostate cancer cells, through inducing pro-apoptotic genes and suppressing the expression of cell cycle-related genes, as well as cancer stemness-associated Wnt/β-catenin pathway [41–43]. Actually, RWF extract is a mixed preparation containing a variety of monoterpenoid indole alkaloids and beta-carboline alkaloids [13, 41, 44]. Among them, alstonine has been reported to possess cytotoxic effects on malignant cells by inducing apoptosis and/or growth arrest [45–48]. It will be interesting to determine whether alstonine, a principal component of the RWF extract used in this study, plays a critical role in ER stress and autophagic apoptosis and whether other alkaloids are also involved. Further dissection of the molecular mechanisms of the anti-BPH activity of RWF extract will be investigated.

## Conclusion

In summary, our findings have demonstrated that RWF extract restrained the development of BPH by activating ER stress and autophagy signal pathway to induce apoptosis in BPH epithelial BPH-1 and stromal WPMY-1 cells, as well as in human BPH samples. This study on RWF extract provided preclinical data that support its potential as an alternative medicine for BPH treatment.

## Supplementary Information

**Additional file 1.**

**Additional file 2.**

## Data Availability

The dataset supporting the conclusions of this article is available in the NCBI GEO Dataset (accession number: GSE151643; hyperlink to the dataset in https://www.ncbi.nlm.nih.gov/geo/query/acc.cgi?acc=GSE151643).

## Abbreviations

3-MA: 3-Methyladenine
4PBA: 4-Phenylbutyrate
BPH: Benign prostatic hyperplasia
CHOP: C/EBP homologous protein
DMSO: Dimethyl-sulfoxide
ER: Endoplasmic reticulum
EIF2α: Eukaryotic translation initiation factor 2 alpha
PARP: Poly (ADP-ribose) polymerase
PDCD4: Programmed cell death 4
PI: Propidium iodide
RWF: *Rauwolfia vomitoria*
SDS-PAGE: Sodium dodecyl sulfate–polyacrylamide gel electrophoresis
