# Supplementary material for: Rauwolfia vomitoria extract suppresses benign prostatic hyperplasia by inducing autophagic apoptosis through endoplasmic reticulum stress
Source: BMC Complement Med Ther. 2022 May 5;22:125. doi: 10.1186/s12906-022-03610-4 (PMC9074266; doi:10.1186/s12906-022-03610-4)
Supplement: Supplementary file 1 — Additional file 1. [file 12906_2022_3610_MOESM1_ESM.docx]

***Supplementary Information***

***Rauwolfia vomitoria* extract suppresses benign prostatic hyperplasia by inducing autophagic apoptosis through endoplasmic reticulum stress**

Guifang Huang^1,2,#^, Xiao He^1,2,#^, Zesheng Xue^3,#^, Yiming Long^2,4,#^, Jiakuan Liu^5^, Jinming Cai^6^, Pengfei Tang^7^, Bangmin Han^6^, Bing Shen^6,7^, Ruimin Huang^1,2,4,*^, Jun Yan^5,*^

1. School of Chinese Materia Medica, Nanjing University of Chinese Medicine, 138 Xianlin Avenue, Nanjing, Jiangsu 210023, China;

2. Shanghai Institute of Materia Medica, Chinese Academy of Sciences, 555 Zuchongzhi Road, Shanghai 201203, China;

3. Model Animal Research Center of Nanjing University, 12 Xuefu Road, Nanjing, Jiangsu 210061, China;

4. University of Chinese Academy of Sciences, No. 19(A) Yuquan Road, Beijing 100049, China;

5. Department of Laboratory Animal Science, Fudan University, 130 Dong’an Road, Shanghai 200032, China;

6. Department of Urology, Shanghai General Hospital, Shanghai Jiao Tong University School of Medicine,100 Haining Road, Shanghai 200080, China;

7. Department of Urology, Shanghai General Hospital of Nanjing Medical University, Shanghai 200080, China

# equally contribution.

*Correspondence: Jun Yan, Ph.D.: yan_jun@fudan.edu.cn;

Ruimin Huang, Ph.D.: rmhuang@simm.ac.cn

**Supplementary Table 1. List of primary antibodies, chemicals, kits, cell lines and reagents**

| **Reagents** | **Company** | **Cat No.** | **Application (dilution)** | **RRID** |  |
| --- | --- | --- | --- | --- | --- |
| ***Antibodies*** | | | | | |
| Actin | Sigma-Aldrich | A2228 | IB (1:5,000) | AB_476697 |  |
| ATF4 | Santa Cruz Biotechnology | sc-390063 | IB (1:1,000) | AB_2810998 |  |
| ATF6 | Cell Signaling Technology | 65880T | IB (1:1,000) | AB_2799696 |  |
| BiP | Cell Signaling | 3177T | IB (1:1,000) | AB_2119845 |  |
| Caspase 3 | Cell Signaling Technology | #9662 | IB (1:1,000) | AB_331439 |  |
| Cleaved Caspase 3 | Cell Signaling Technology | #9661 | IB (1:1,000) | AB_2341188 |  |
| CHOP | Cell Signaling Technology | #2895 | IB (1:1,000) | AB_2089254 |  |
| eIF2α | ABclonal | A0764 | IB (1:1,000) | AB_2757387 |  |
| p-eIF2α(Ser51) | Cell Signaling Technology | 3398T | IB (1:1,000) | NA |  |
| LC3 | Cell Signaling Technology | #3868 | IB (1:1,000) | AB_2137707 |  |
| p62 | Proteintech | 18420-1-AP | IB (1:1,000) | AB_10694431 |  |
| PARP | Cell Signaling Technology | #9532 | IB (1:1,000) | AB_659884 |  |
| Cleaved PARP | Cell Signaling Technology | #5625 | IB (1:1,000) | AB_10699459 |  |
| PDCD4 | Proteintech | 12587–1-AP | IB (1:1,000) | AB_2162296 |  |
| PERK | Cell Signaling Technology | 5683T | IB (1:1,000) | AB_10841299 |  |
|  | Santa Cruz Biotechnology | sc-377400 | IB (1:1,000) | AB_2762850 |  |
| p-PERK(Thr980) | BIOSS | bs-3330R | IB (1:1,000) | AB_10855345 |  |
| ULK2 | ABclonal | A15244 | IB (1:1,000) | AB_10787100 |  |
| ***Chemicals*** | | | | | |
| 3-Methyladenine (3-MA) | MCE | HY-19312 |  | NA |  |
| 4-phenyl butyrate (4-PBA) | MCE | HY-A0281 |  | NA |  |
| Cisplatin | MCE | HY-17394 |  | NA |  |
| DTT | Sangon Biotech | A620058-0025 |  | NA |  |
| Hydrocortisone | Sigma-Aldrich | H0135 |  | NA |  |
| MTT | Biofroxx | 143315 |  | NA |  |
| Polyethylenimine (PEI) | Sigma-Aldrich | 408727 |  | NA |  |
| Polybrene | Sigma-Aldrich | TR-1003 |  | NA |  |
| Puromycin | Selleck | 58-58-2 |  | NA |  |
| RWF extract | Maison Beljanski | NA |  | NA |  |
| Trizol | Life Technologies | 15596018 |  | NA |  |
| ***Kits*** | | | | | |
| FITC Annexin V Apoptosis Detection Kit I | BD Biosciences | #556547 |  | NA |  |
| Hifair II 1st strand cDNA Synthesis SuperMix | YEASEN | 11123ES60 |  | NA |  |
| **Supplementary Table 1. List of primary antibodies, chemicals, kits, cell lines and reagents (Cont’d)** | | | | | |
| **Reagents** | **Company** | **Cat No.** | **Application (dilution)** | **RRID** |  |
| ***Kits (Cont’d)*** | | | | | |
| SuperSignal west femto maximum sensitivity substrate | Thermo Fisher Scientific | 34095 |  | NA |  |
| ***Plasmid*** | | | | | |
| pLVX-GFP-LC3 | Gift from Dr. Jin Ren (Shanghai Institute of Materia Medica, CAS) | NA |  | NA |  |
| pMD2.G packaging plasmid | In the lab | NA |  | NA |  |
| psPAX2 envelope plasmid | In the lab | NA |  | NA |  |
| ***Cell line*** | | | | | |
| BPH-1 | Gift from Dr. Simon Hayward (Vanderbilt University Medical Center) | NA |  | NA |  |
| WPMY-1 | The Cell Bank of Type Culture Collection of Chinese Academy of Sciences | [GNHu36](https://www.cellbank.org.cn/search-detail.php?id=321) |  | CVCL_3814 |  |
| ***Culture medium and reagents*** | | | | | |
| Antibiotic/antimycotic solution | BasalMedia | S120JV |  | NA |  |
| DMEM medium | Corning | 10-013-CV |  | NA |  |
| DMEM/F-12 medium | Corning | 10-920-CV |  | NA |  |
| Fetal bovine serum (FBS) | Life Technologies | 10091-148 |  | NA |  |
| Insulin | Sigma-Aldrich | I1882 |  | NA |  |
| Millex-HV syringe filter unit, low adsorption 0.45 μM | MERCK | SLHU033RB |  | NA |  |
| Penicillin/streptomycin | BasalMedia | S110JV |  | NA |  |
| RPMI1640 medium | Corning | 10-040-CV |  | NA |  |
| Sterile gelatin sponge | Hushida Medical Care Technology Co. Ltd. | HSD-B |  | NA |  |

**Supplementary Table 2. List of primer sequences for qRT-PCR**

| **Gene symbol** | **Direction** | **Primer sequence (5’- -3’)** |
| --- | --- | --- |
| *ACTB* | Forward | CAGAGCCTCGCCTTTGCCGATC |
|  | Reverse | CATCCATGGTGAGCTGGCGGCG |
| *ATF4* | Forward | tcatgggttctccagcgaca |
|  | Reverse | gtcatctggcatggtttcca |
| *DDIT3/CHOP* | Forward | CCTTTCTCCTTCGGGACACT |
|  | Reverse | CTCTGGGAGGTGCTTGTGAC |
| *EIF2AK3/PERK* | Forward | taaaggactgatgcacaggg |
|  | Reverse | ctcatatacagtttggtccc |
| *PDCD4* | Forward | TCTGGGAAAGGAAGGGGACT |
|  | Reverse | TGCCAACACTGGTACTCCAC |
| *SQSTM1/p62* | Forward | AAGCCGGGTGGGAATGTTG |
|  | Reverse | CCTGAACAGTTATCCGACTCCAT |
| *ULK2* | Forward | GTGGTATTCGCATCAAAATAGCG |
|  | Reverse | CACAAGTCAGCCTTAGCATCATA |

**Supplementary Table 3. List of primer sequences for RT-PCR**

| **Gene symbol** | **Direction** | **Primer sequence (5’- -3’)** |
| --- | --- | --- |
| *ACTB* | Forward | CTGGGACGACATGGAGAAAA |
|  | Reverse | AAGGAAGGCTGGAAGAGTGC |
| *XBP1* | Forward | GAATGAAGTGAGGCCAGTGG |
|  | Reverse | ACTGGGTCCTTCTGGGTAGA |
