# Supplementary material for: Rauwolfia vomitoria extract suppresses benign prostatic hyperplasia by inducing autophagic apoptosis through endoplasmic reticulum stress
Source: BMC Complement Med Ther. 2022 May 5;22:125. doi: 10.1186/s12906-022-03610-4 (PMC9074266; doi:10.1186/s12906-022-03610-4)

**Raw data on Western blotting and agarose electrophoresis**

***Rauwolfia vomitoria* extract suppresses benign prostatic hyperplasia by inducing autophagic apoptosis through endoplasmic reticulum stress**

Guifang Huang^1,2,#^, Xiao He^1,2,#^, Zesheng Xue^3,#^, Yiming Long^2,4,#^, Jiakuan Liu^5^, Jinming Cai^6^, Pengfei Tang^7^, Bangmin Han^6^, Bing Shen^6,7^, Ruimin Huang^1,2,4,*^, Jun Yan^5,*^


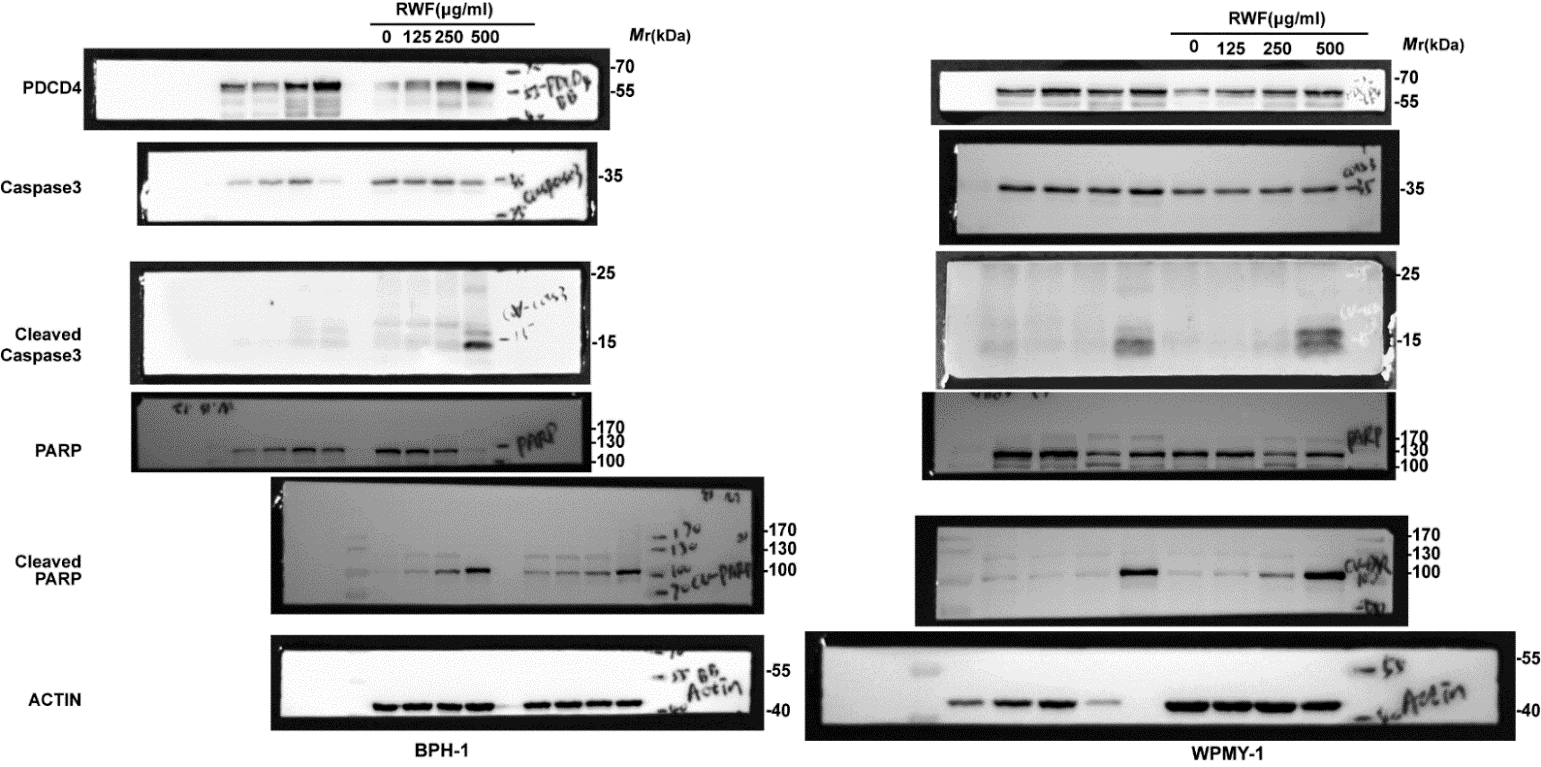


**Figure 2D**

**Figure 3D**


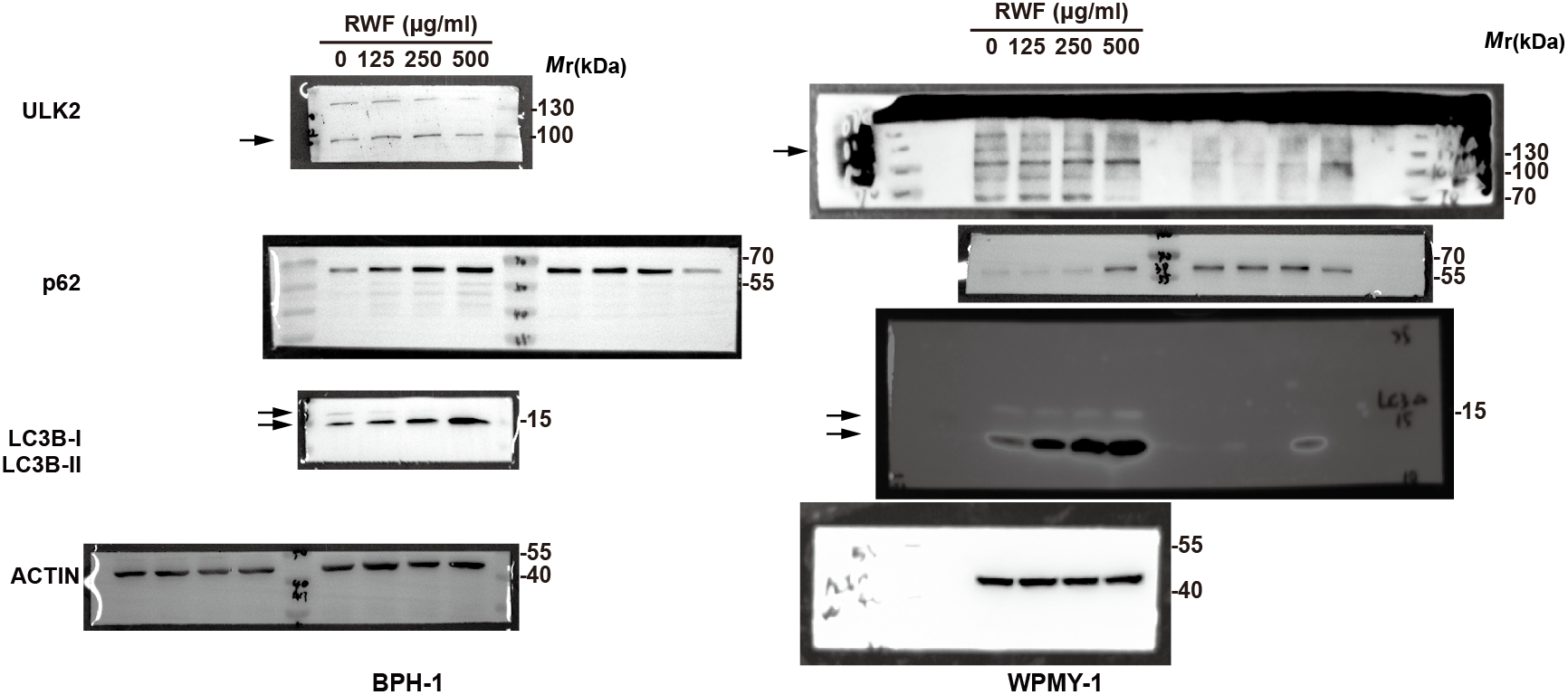


**Figure 4F**

**
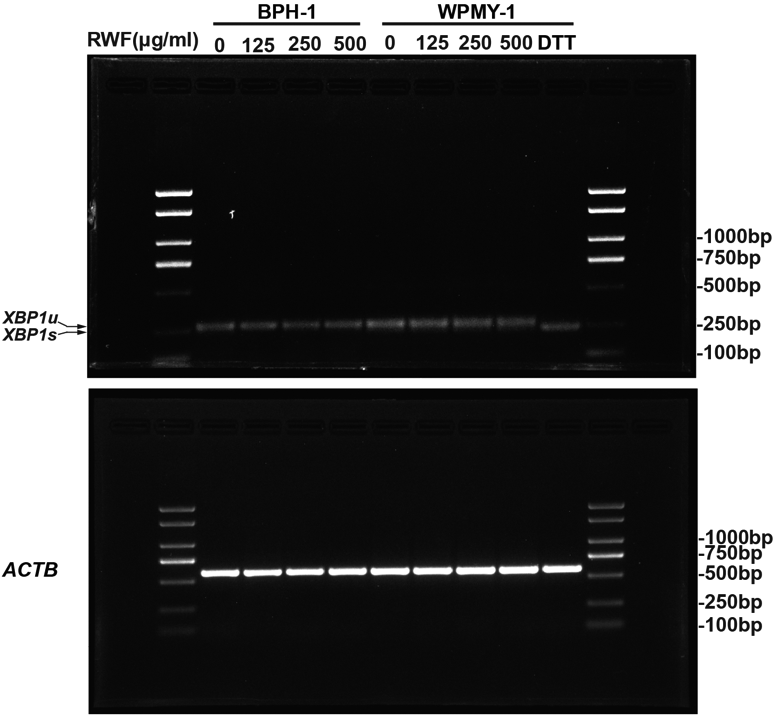
**

**Figure 4G**


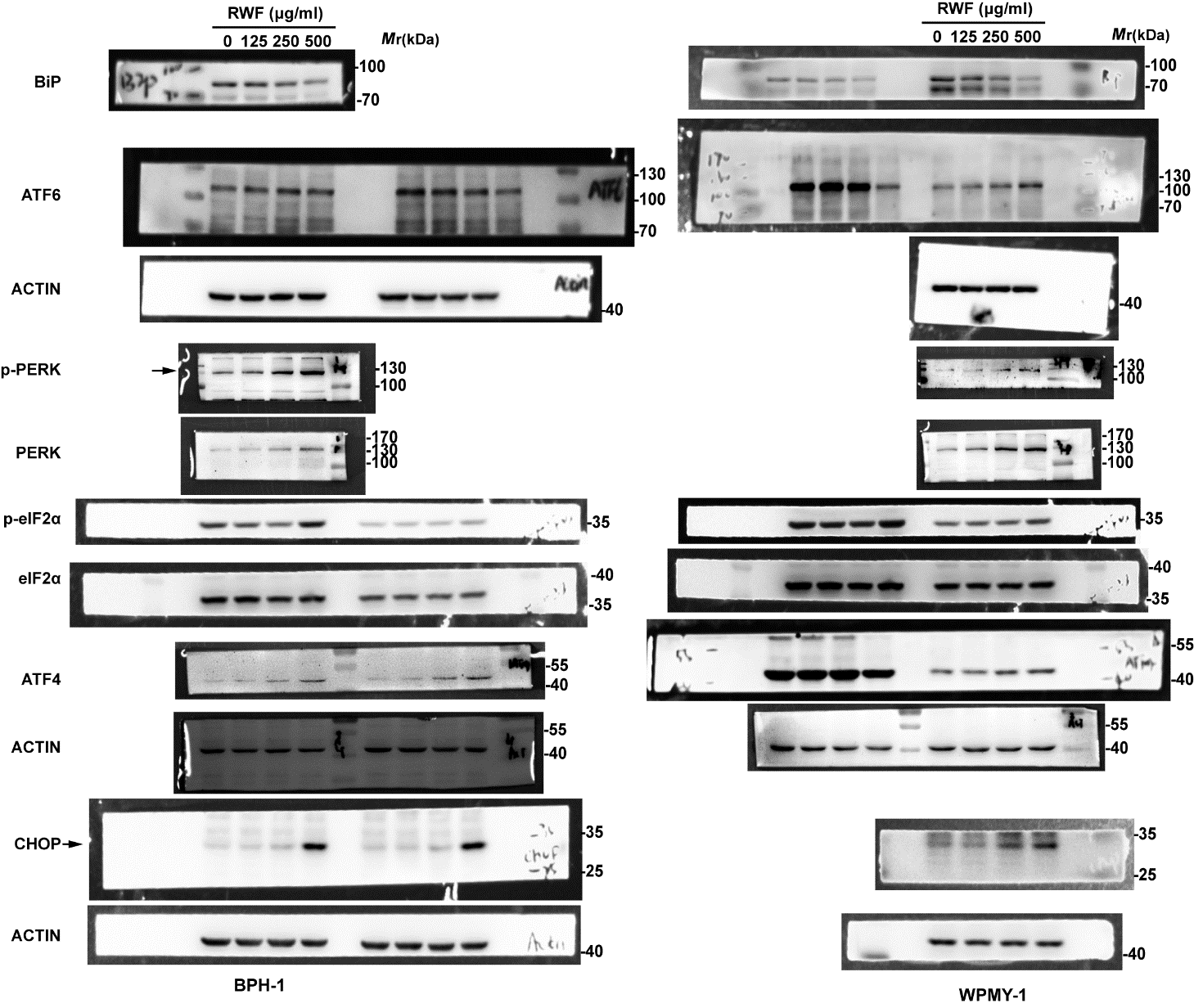


**Figure 5A**


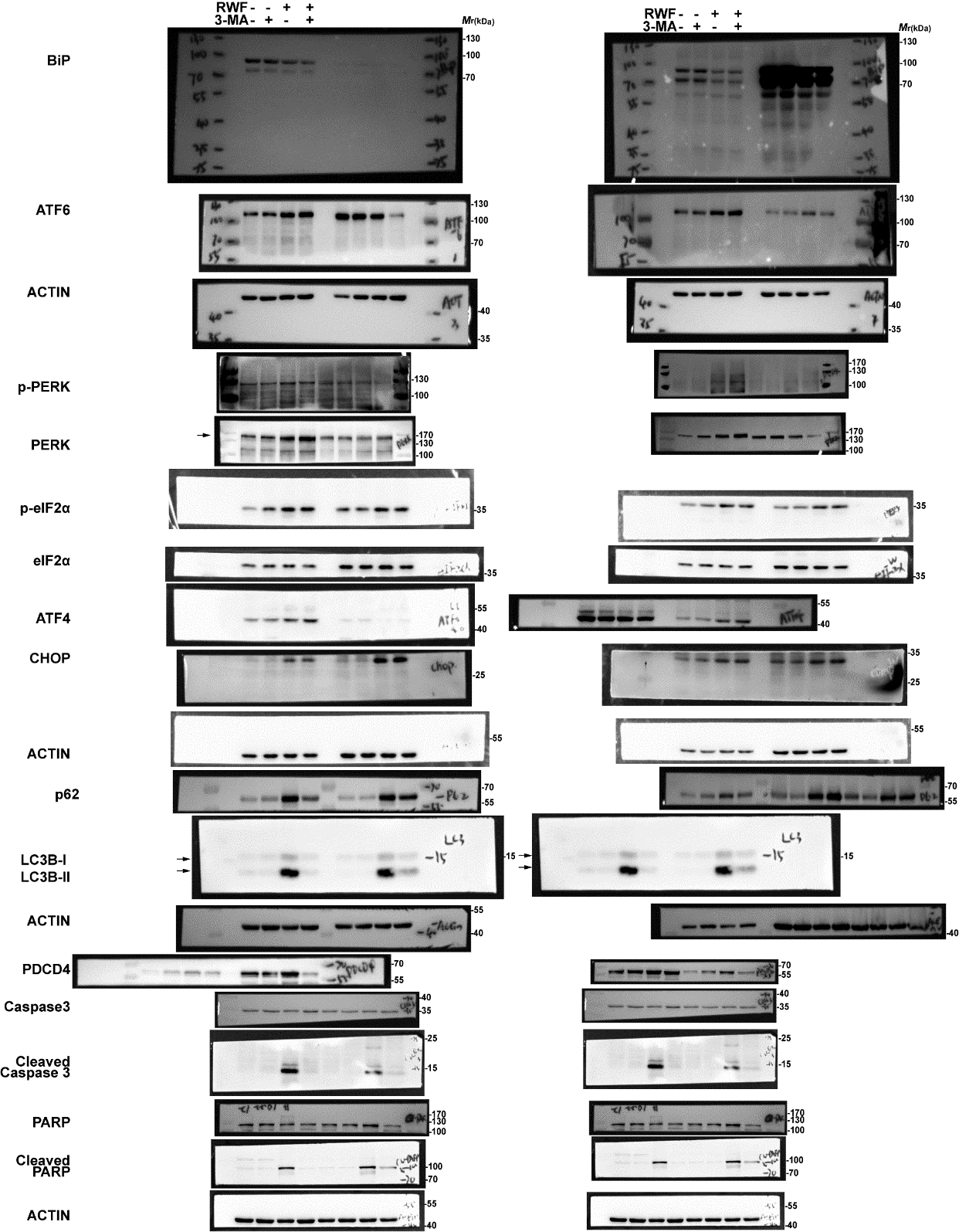


**Figure 5D**


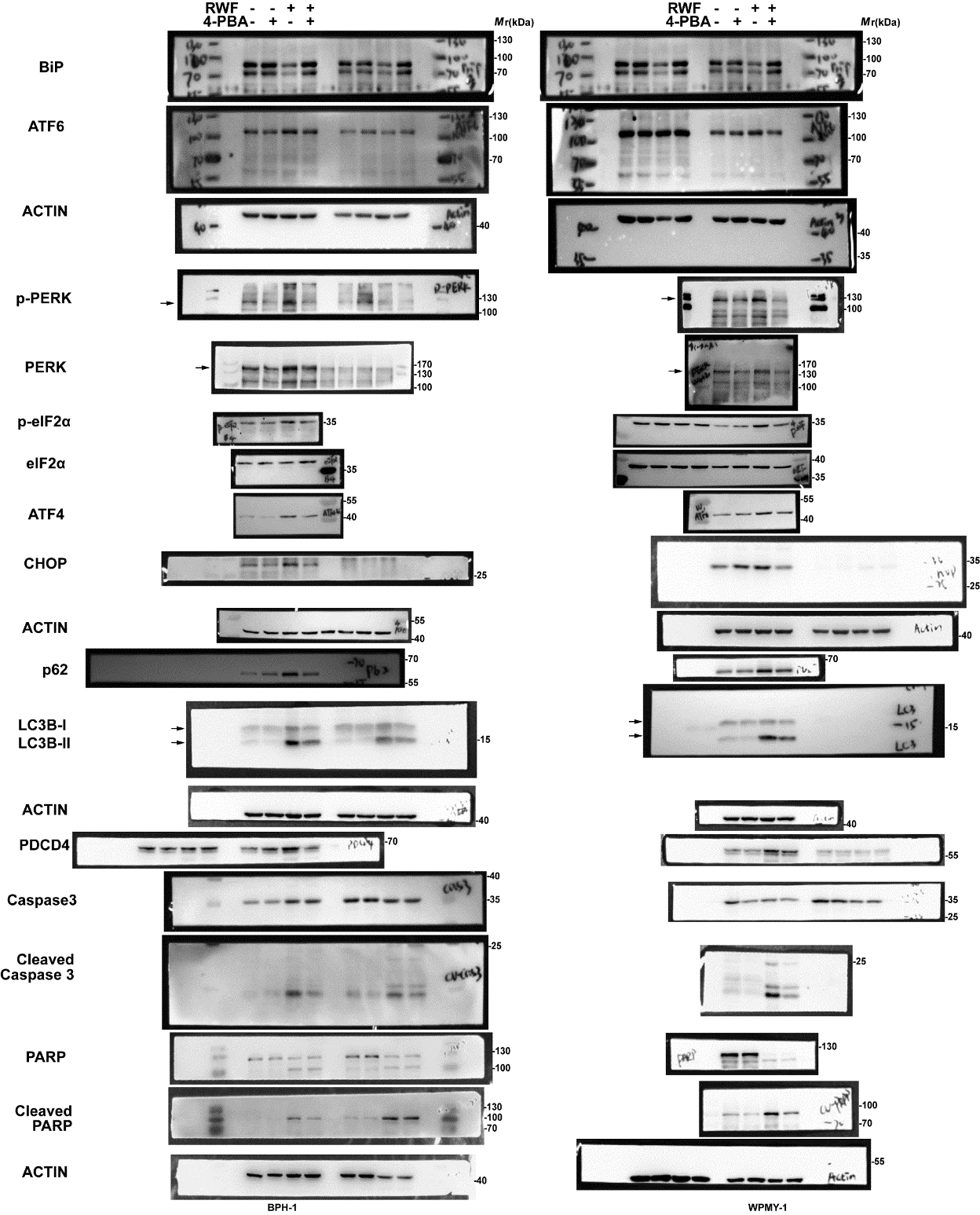


**Figure 6C**


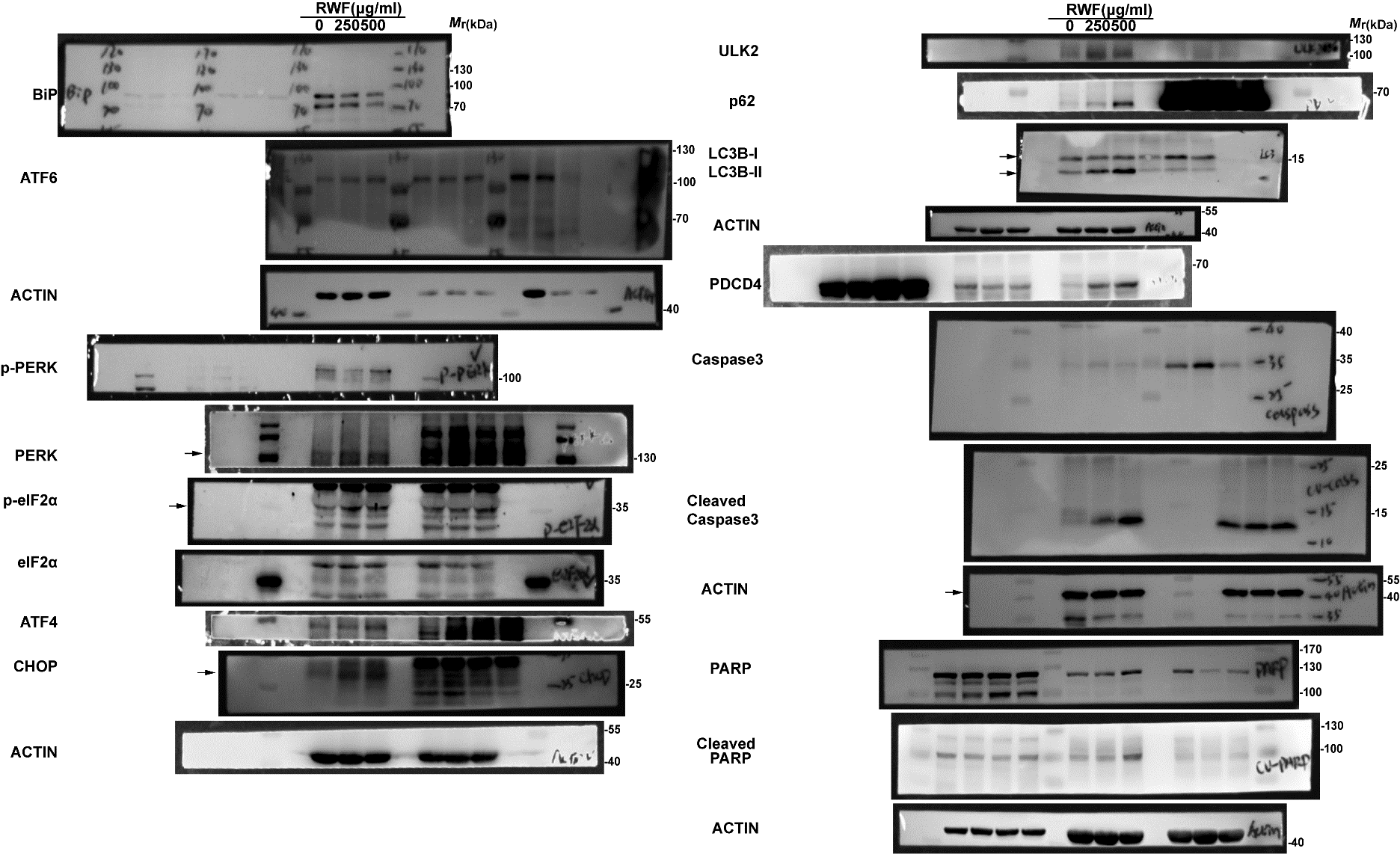

Supplement: Supplementary file 2 — Additional file 2. [file 12906_2022_3610_MOESM2_ESM.docx]
